# Biomarkers of Brain Injury in the Premature Infant

**DOI:** 10.3389/fneur.2012.00185

**Published:** 2013-01-22

**Authors:** Martha Douglas-Escobar, Michael D. Weiss

**Affiliations:** ^1^Department of Pediatrics, University of FloridaGainesville, FL, USA; ^2^McKnight Brain Institute, University of FloridaGainesville, FL, USA

**Keywords:** biomarkers, intraventricular hemorrhage, post-hemorrhagic ventricular dilation, periventricular leukomalacia, brain injury

## Abstract

The term “encephalopathy of prematurity” encompasses not only the acute brain injury [such as intraventricular hemorrhage (IVH)] but also complex disturbance on the infant’s subsequent brain development. In premature infants, the most frequent recognized source of brain injury is IVH and periventricular leukomalacia (PVL). Furthermore 20–25% infants with birth weigh less than 1,500 g will have IVH and that proportion increases to 45% if the birth weight is less than 500–750 g. In addition, nearly 60% of very low birth weight newborns will have hypoxic-ischemic injury. Therefore permanent lifetime neurodevelopmental disabilities are frequent in premature infants. Innovative approach to prevent or decrease brain injury in preterm infants requires discovery of biomarkers able to discriminate infants at risk for injury, monitor the progression of the injury, and assess efficacy of neuroprotective clinical trials. In this article, we will review biomarkers studied in premature infants with IVH, Post-hemorrhagic ventricular dilation (PHVD), and PVL including: S100b, Activin A, erythropoietin, chemokine CCL 18, GFAP, and NFL will also be examined. Some of the most promising biomarkers for IVH are S100β and Activin. The concentrations of TGF-β1, MMP-9, and PAI-1 in cerebrospinal fluid could be used to discriminate patients that will require shunt after PHVD. Neonatal brain injury is frequent in premature infants admitted to the neonatal intensive care and we hope to contribute to the awareness and interest in clinical validation of established as well as novel neonatal brain injury biomarkers.

## Introduction

Increasing rates of survival of extremely premature infants has produced a shift of paradigms from “survival” to “prevention of morbidity” including brain injury. The discovery and validation of neonatal biomarkers of brain injury is a key step in the evolution of neonatal neuroprotection. These markers may enable the clinicians to screen infants for brain injury, monitor the progression of disease, identify injured brain regions, and assess the efficacy of neuroprotective strategies procedures in clinical trials. Currently, clinicians do not use biomarkers to care for neonates with brain injuries. This review will examine potential biomarkers for the most common brain injuries in premature infants, such as intraventricular hemorrhage (IVH), post-hemorrhagic hydrocephalus, and periventricular leukomalacia (PVL).

## Biomarkers of Intraventricular Hemorrhage

One major source of long-term neurologic deficits in premature neonates is the injury to the germinal matrix and the subventricular zone (Volpe, 2009). These injuries produce IVH. Approximately 20–25% of premature infants weighing less than 1,500 g will have an IVH (Volpe, 2008). The risk of IVH is inversely related to gestational age and birth weight. Forty-five percent of infants weighing 500–750 g develop IVH (Wilson-Costello et al., 2005). Immature blood vessels in the germinal matrix, a highly vascular region of the brain, combined with poor tissue vascular support, predispose premature infants to hemorrhage (Volpe, 2009). Clinical presentation of IVH can range from an acute newborn deterioration of the newborn (with apnea, pallor, acidosis, hypotension, bulging fontanel, seizures, and decreased muscle tone) to a “clinically silent syndrome” (no symptoms). Biomarkers that hold promise in predicting which neonates may suffer an IVH and long-term deficits will be reviewed.

*The protein S100 family* encompasses many calcium sensor proteins that modulate biological activity via calcium binding (Ikura, 1996). In particular, *S100β* (a homodimer of the subunit beta) protein is primarily synthesized in the brain by astrocytes and is quickly released from the brain into the blood when the blood-brain-barrier is disrupted (Kapural et al., 2002; Marchi et al., 2003). In the central nervous system S100β protein is predominantly concentrated in the astroglial cell population (Heizmann, 1999). However, reports of extra cranial sources of S100β, especially from adipose and muscle tissue, may confound its interpretation in the clinical setting (Otto et al., 2000; Bloomfield et al., 2007). S100β has a dual function depending on its concentrations. At nanomolar physiological concentrations, S100β is neurotrophic (Haglid et al., 1997). However, when S100β is overexpressed (in micromolar concentrations), it enhances neuroinflammation and neuronal apoptosis (Van Eldik and Wainwright, 2003). Recently an excess of S100β and amyloid precursor protein has been linked to impaired neurogenesis (due to the gliocentric shift of neural progenitor cells) in Down syndrome (Bouvier et al., 2011; Lu et al., 2011).

S100B has been well studied in the pediatric population. Immunoassay kits are commercially available and can detect S100β in many biological fluids such as urine, blood, CSF, amniotic fluid, saliva, and milk (Gazzolo and Michetti, 2010; Gazzolo et al., 2010). Furthermore, reference ranges are available for the pediatric population including preterm and term healthy newborns (Gazzolo et al., 2007; Bouvier et al., 2011). In general, healthy children have higher serum S100β concentrations than adults and the concentrations decline over time, especially during the first 6 months of life (Bouvier et al., 2011). Similarly, urinary S100β concentrations are higher in premature infants than in term newborns and steadily decrease with advancing gestational age (Gazzolo et al., 2007).

Gazzolo et al. (2006) reported that maternal blood concentrations of S100β >0.72 mcg/L were able to predict neonatal IVH with 100% sensitivity, 99% specificity, and 0.999 area under the ROC curve. However, measurements of serum S100β during pregnancy could be affected by multiple factors, such as gestational age, intrauterine growth, prenatal steroids use, twin gestation, and trisomy 21 (Gazzolo et al., 2003a,b; Sannia et al., 2010).

Premature infants with high concentrations of S100β in urine have higher mortality than matched controls for gestational age and weight with a positive predictive value of 78% and a negative predictive value of 100% (Gazzolo et al., 2005). S100β also plays a role in predicting IVH in neonates. Newborns that developed IVH have elevated S100β concentrations in blood and urine (Gazzolo et al., 1999, 2001). In addition, the urine S100β level correlates with the degree of IVH (Gazzolo et al., 2001). Taken together, these publications support the hypothesis that early brain injury may be responsible for a continuous release of S100β protein from the CNS into the systemic circulation and urine. Because IVH is more frequent in very small infants with birth weight 500–750 g, biomarkers that do not require blood samples are more clinical relevant. Therefore, one major advantage of S100β is that it can be measured in urine. In addition, there are commercially available kits to measure S100β. We believe that the next step in the validation of S100β as biomarker of IVH would be studies that incorporate its association with brain injury assessed by brain MRI and long-term functional outcomes.

*Activin* is another proposed biomarker for IVH. Activin, a member of the transforming growth factor-β superfamily, is a trophic factor that regulates differentiation and proliferation of neurons and a wide variety of cells (Florio et al., 2007). Activin receptors are highly expressed in neuronal cells and neuronal activity up-regulates activin mRNA expression (He et al., 2012). In animal models, Activin is neuroprotective during excitotoxic brain injury (Mukerji et al., 2007). In transgenic mice, activin regulates spine formation, behavioral activity, anxiety, adult neurogenesis, late-phase long-term potentiation, and the maintenance of long-term memory (Ageta et al., 2008; Zheng et al., 2009). Florio et al. (2006) found that premature newborns that developed IVH had high concentrations of Activin A in blood samples drawn during their first hour of life. In his cohort of 53 infants <32 weeks gestational age, 21% developed IVH detected by serial head ultrasound (HUS). Activin above 0.8 mcg/L predicted IVH with 100% sensitivity of 100%, and 93% specificity (with positive predictive value of 79%). Activin A is also increased in term newborns with moderate or severe asphyxia suggesting that activin is released after neuronal (Florio et al., 2004). Activin A should be validated in larger cohort of premature infants and correlated not only with IVH diagnosed by HUS but also with term corrected brain MRI (a more sensitive and specific brain injury detection).

*Erythropoietin* (EPO) is also a potential biomarker of IVH. EPO and its receptor are expressed in astrocytes, neurons, and endothelial brain cells (Marti, 2004). Bhandari published a prospective pilot cohort study of cord blood concentration of EPO in 116 infants less than 34 weeks GA (Bhandari et al., 2011). In this study, 25% infants had IVH diagnosed by serial HUS. Elevated cord blood EPO levels were predictors of IVH even after correction for gestational age. In the same cohort, inflammatory markers (such cord blood IL-6, pH, and early onset of neonatal sepsis) were not associated with IVH. These results suggest that elevated cord blood EPO may predict neonatal risk for IVH, independent of fetal inflammatory status. EPO production is increased in response to fetal hypoxia (Davis et al., 2003; Teramo and Widness, 2009). Thus, elevated EPO in cord blood may indicate fetal hypoxic conditions that lead to injury of the germinal matrix resulting in IVH. EPO is attractive as a biomarker because it can be measured at birth and the results are available the same day. Nevertheless it is important to establish if high levels Epo correlate with functional outcomes.

*Chemokine ligand 18 (CCL18)* belongs to the CC-chemokine family, is encoded in chromosome 17q11.2 and participates in the lymphocytes homing and the primary immune response (Zlotnik et al., 2006). As a result, inflammatory conditions may increase levels of CCL18 (Schutyser et al., 2005). Preterm infants who developed cerebral palsy have lower cord blood levels of CCL18 (Kaukola et al., 2004). Patients with traumatic brain injuries have elevated CCL18 in biopsies of brain tissue (Chang et al., 2010). In a prospective cohort study of 163 premature infants (less than 32 weeks of gestation), Kallankari et al. (2010) analyzed 107 cord blood immunoproteins, 12 cytokines from the peripheral blood, serial HUS in all newborns, and brain immunohistochemistry of chemokine receptors from the autopsies of 14 patients. Cord chemokine CCL18 robustly predicted the risk of IVH grades II-IV and was not associated with chorioamnionitis or funisitis. CCL18 receptor was detectable in the choroid plexus, periventricular capillary endothelium, ependymal cells, and the germinal matrix which may explain that high cord levels of CCL18 could be protective against IVH and brain injury by blocking the action of agonistic ligands on CCR3, thereby inhibiting leukocyte degranulation and inflammatory activation. Because CCL18 is an inflammatory mediator, it may be a very sensitive but not specific biomarker for IVH.

*Uric acid* (UA) is the end product of purine metabolism. Because UA has poor solubility, continuous renal excretion is necessary to avoid its toxic accumulation. High serum UA concentrations are expected when there are increase in production or decrease in its excretion. During hypoxic-ischemic events, hypoxanthine, a purine intermediate metabolite, accumulates. During the reperfusion states, hypoxanthine is then converted to UA (Perlman and Risser, 1998). Perlman and Risser (1998) reported elevated concentrations of UA (during first 24 h) in premature infants that developed IVH and PVL. In support of these findings, Aliefendioglu et al. (2006) found that high UA concentrations in CSF were associated with a higher risk of IVH. However, other study of low birth weight infants did not find association between elevated UA in serum and IVH (Sysyn and Rozycki, 2003). UA has conflicting results as brain injury biomarker.

*Brain-type creatine phosphokinase (CPK-BB)* is an enzyme expressed in various cell types and catalyzes the conversion of creatine to phosphocreatine (energy reservoir for cells that consume ATP rapidly). Van de Bor et al. (1988) found that higher serum CPK-BB during the first day of life was associated with IVH detected by serial HUS. Nevertheless, the small number of newborns with severe IVH limited the interpretation of the results of this pilot study. In a later study, Amato et al. (1989) found that only the CPK-BB values obtained during the first 6 h of life, but not later, were associated with IVH. This early elevations of CPK-BB concentration suggest association with prenatal events. One plausible explanation is that during hypoxic-ischemic events, brain cells deplete their ATP and increase CPK-BB concentrations to obtain more phosphocreatine (other source of cell energy). In our opinion, this biomarker shows limited predictability for brain injury because it is not specific to brain injury and is only increased for few hours.

*IL-6 and C-reactive protein (CRP)* are inflammatory markers, therefore non-specific markers of brain injury. Sorokin et al. followed a cohort of 475 asymptomatic pregnant women at risk for preterm birth. He found that high maternal serum concentrations of IL-6 and CRP were associated with increase risk of IVH in their premature infants even after adjusting for gestational age (Sorokin et al., 2010). Notably, 25 out of 30 neonates had grade I IVH. It is possible maternal inflammatory markers reflect the fetal environment and therefore could be associated with brain pathology but they are not specific for brain injury.

## Biomarkers for Post-Hemorrhagic Ventricular Dilation

Following a large IVH, blood clots throughout the ventricular system may block the channels for the reabsorption of cerebrospinal fluid and the lateral ventricles enlarge producing Post-hemorrhagic ventricular dilation (PHVD; Whitelaw and Aquilina, 2012). Overtime, the chronic inflammation, free iron, free radicals, and the excessive intracranial pressure produce not only ventricle enlargement but also progressive periventricular white matter injury. Patients with PHVD have worse outcomes: 40% develop cerebral palsy and 25% have multiple impairments (Ventriculomegaly trial group, 1994; Kennedy et al., 2001). Patients with large amounts of blood clots in the ventricles have a higher risk for shunt placement (Whitelaw, 2001; Whitelaw and Aquilina, 2012). Deciding when to intervene in patients with PHVD could be a challenge in part due to the small size of the patient (often between 1 and 2 kg) and complications related to drains. There is no precise test that could help to determine when is the best time to place a drain (external drain or shunt) to avoid secondary periventricular brain damage (Davies et al., 2000; Whitelaw and Aquilina, 2012). The following biomarkers are associated with PHVD and need for shunt:

*Transforming growth factor beta 1 (TGF-β1)* is released into CSF after intraventricular bleeding and up-regulates the genes to increase production of extracellular matrix (ECM) proteins such as collagen and fibronectin (Whitelaw and Aquilina, 2012). Excessive production of ECM could lead to blockage of CSF reabsorption. Therefore, TGF β1 could serve as a biomarker of PVHD. Whitelaw et al. (1999) found higher concentrations of TGF β1 in CSF of preterm infants with PHVD than preterm controls. Among the PHVD patients, those with highest TGF β1 concentrations had higher rate of shunt placement. TGFβ1 > 6.5 ng/mL in CSF was 80% sensitive and 78% specific to discriminate which infants with PHVD would require a shunt. By contrast, Heep et al. (2004) found that increased concentrations of TGFβ-1 in CSF did not correlate with PHVD but correlated with white matter injury. Lipina et al. (2010) reported that patients with PVHD with TGF β-1 > 2,396 pg/mL had a sensitivity of 79% and specificity of 80% to predict which patients would not benefit of endoscopic third ventriculostomy (surgery that allow CSF to leave the ventricular system) and need shunt placement. Elevated TGF β-1 is associated with worse course of PHVD either because patient has higher risk of shunt placement or white matter injury. Unfortunately, this biomarker is only measured in CSF and need validation in larger studies.

*Transforming growth factor beta-2 (TGF-β2)* is associated with a decreased proliferation of neuronal precursors and induction of cell death of oligodendrocytes. Chow et al. (2005) found that in patients with PHVD, TGF β-2 in CSF was 20 times greater if patients required shunt and it was associated with worse neurodevelopment outcome at 15 months. It is possible that high concentrations of TGF-β2 in CSF correlate with worse prognosis due to its effects with neurons and oligodendrocytes. This potential biomarker needs validation in larger cohort of patients.

*Proteins of the matrix metallo-proteinases (MMPs)* family are involved in the breakdown of ECM proteins (Rosell et al., 2008). Okamoto et al. (2008) found that *MMP-9* in CSF was higher in patients with PHVD than controls. Higher MMP-9 concentrations possible reflect ongoing brain tissue remodeling in patients with PHVD. Patients with PHVD that required a shunt had higher MMP-9 levels than controls but lower MMP-9 concentrations than PHVD patients without need for a shunt. A plausible explanation for this finding is that patients with lower MMP-9 concentrations could not degrade the amount of extracellular proteins produced and end with CSF outflow obstruction requiring shunt placement. Validation studies are necessary for this pilot report.

*Plasminogen activator inhibitor 1 (PAI-1)* is one of the main inhibitors of fibrinolysis (physiological breakdown of blood clots; Booth et al., 1988). Hansen et al. showed that CSF concentration of PAI-1 was highest in patients with PHVD compared with neonates with IVH without PHVD (Hansen et al., 1997, 2000). It is possible that neonates who develop IVH and have impaired blood breakdown due to high concentrations of PAI-1 would develop PHVD. This is promising biomarkers but needs more validation studies.

Whitelaw et al. (2001) found that *median neurofilament (NFL) and glial fibrillary acidic protein (GFAP)* concentrations in infants with PHVD were 20–200 times higher than controls. In the same study, patients with PHVD had four times higher S100 protein in CSF than control patients and GFAP concentrations correlated with death or disability. We think that NFL and GFAP are plausible biomarkers of PHVD but they required CSF samples. Again, this is promising pilot data but needs confirmation with large number of patients.

## Biomarkers of Periventricular Leukomalacia

Periventricular leukomalacia is a cerebral white matter injury that occurs to some degree in 50% of neonates with birth weights less than 1,500 g (Volpe et al., 2011). PVL is associated with a decrease in the volume of the cortex, thalamus, and basal ganglia (Volpe et al., 2011). This injury likely account for 90% of the neurologic deficits, including cerebral palsy, cognitive, behavioral, and attention deficits, that occurs in surviving premature neonates. In addition, up to 50% of neonates with congenital heart disease requiring surgery acquire PVL (Galli et al., 2004).

Diagnosis of PVL has been limited to later stages of PVL where radiological changes are visible in ultrasound or brain MRI. There is a paucity of publications on PVL serum biomarkers. Recent research done on autopsy of premature newborns with PVL has lead to the discovery of immunomarkers of early stages of PVL and could change our understanding of its physiopathology and prevalence. Some of the new described tissue biomarkers of PVL are:

*Human beta-amyloid precursor protein (β-APP)* is marker of wide diffuse axonal damage for early stages of PVL (Arai et al., 1995; Hirayama et al., 2001) and *Fractin* that is an apoptotic marker (Haynes et al., 2008). β*-*APP and fractin could improve understand the pathogenesis of diffuse axonal damage in PVL, including whether or not this damage results in irreversible axonal loss and impaired neuronal function. There is a paucity of publications on biomarkers of PVL, an area on increasing interest due to its high frequency in extremely premature neonates and infants with congenital heart disease.

## Summary

We found that some of the most promising biomarkers for IVH are S100β and Activin. PHVD biomarkers like TGF-β1, MMP-9, and PAI-1, could be used to discriminate patients that will require shunt. We summarized the characteristic of the biomarkers and its potential usefulness in Tables 1 and 2. There is a paucity of publications that validated the potential biomarkers of brain injury with more accurate brain damage assessment, such as brain MRI. In addition potential biomarkers should explore their correlation with functional brain outcomes such as amplitude integrated EEG, full EEG, functional brain MRI, and long term neurodevelopmental follow-up. The available data indicate that such studies are not only justified but also urgently needed to care for newborns, especially those with extreme prematurity.

**Table 1:** **Summary of biomarkers characteristics**.

Biomarker	Description	Cell specificity	Pathophysiology
S100β	Protein that binds calcium and is a major component of the cytosol in various cell types (Ikura, 1996)	*Astroglial* cells have a high concentration of S100β (Heizmann, 1999). Other cells can release S100β (Bloomfield et al., 2007)	Increased concentrations of S100β occur predominantly after astrocyte death (Van Eldik and Wainwright, 2003)
Activin A	Trophic factor, member of the transforming growth factor-β superfamily (Florio et al., 2007)	Activin receptors are highly expressed in *neuronal* cells (Florio et al., 2007)	Increased concentrations of Activin A occur predominantly after neuronal injury (Florio et al., 2006, 2007)
Erythropoietin	Trophic factor and is synergistic with other growth factors (Marti, 2004)	Produced mainly by interstitial fibroblasts in the kidneys and placenta and hepatocytes in the fetus (Davis et al., 2003). EPO and its receptor are expressed throughout the brain in *glial cells, neurons*, and *endothelial cells* (Marti, 2004)	Increased concentrations of EPO occur after hypoxic conditions (endogenous mechanism neuronal protection; Marti, 2004; Teramo and Widness, 2009)
Chemokine CCL18	Member of the CC-chemokine family (Zlotnik et al., 2006)	Monocytes and dendritic cells secrete CCL18. CCL18 receptor is detectable in the choroid plexus, periventricular capillary endothelium, ependymal cells, and the *germinal matrix* (Kallankari et al., 2010)	High concentrations of CCL18 blocks the action of agonistic ligands on CCR3 (decreasing inflammatory response) and could be protective factor for IVH (Chang et al., 2010; Kallankari et al., 2010)
TGF-b1	Member of the transforming growth factor-β superfamily (Pal et al., 2012)	Main sources of TGF-β1 in the injured brain are *astrocytes* and *microglia* but *neurons* can produce it as well (Heinemann et al., 2012; Pal et al., 2012). TGF-β1 released into CSF after IVH, up-regulate the genes for *extracellular matrix* (Whitelaw and Aquilina, 2012)	High concentrations of TGF- (β1 may trigger excessive production of ECM leading to blockage of CSF reabsorption, therefore could serve as biomarker of PVHD (Whitelaw et al., 1999)
MMP-9	Member of the proteins of the matrix metallo-proteinases (MMPs) family (Rosell et al., 2008; Pal et al., 2012)	*All cell types of the CNS* are potential sources of MMPs. MMP-9 is involved in the breakdown of *extracellular matrix* proteins (Okamoto et al., 2008; Rosell et al., 2008)	Higher concentrations of MMP-9 are needed to degrade the extracellular proteins after IVH. Lower MMP-9 concentrations in CSF of patients with PVHD could predict patients that will need shunt (Okamoto et al., 2008)
PAI-1	Main inhibitor fibrinolysis (Booth et al., 1988)	PAI is mainly produced by *vascular endothelial cells*, but also secreted by many other tissues (hepatic, adipose, etc.). This protein that *inhibit* tissue plasminogen activator and urokinase, the *activators of plasminogen* (Booth et al., 1988)	High concentrations of PAI-1 in CSF could impaired blood removal (fibrinolysis) after IVH, leading to PVHD (Hansen et al., 1997, 2000)
GFAP and NFL	Cytoskeletal intermediate and median filament protein found in the astrocytes (Mayer et al., 1989; Eng and Ghirnikar, 1994; Middeldorp and Hol, 2011)	Specific marker of *differentiated astrocytes* (Middeldorp and Hol, 2011)	Higher concentrations of GFAP and NFL in CSF are expected after astrocyte death (Whitelaw et al., 2001)

*Summary of potential biomarkers of brain injury including intraventricular hemorrhage (IVH), and post-hemorrhagic ventricular dilation (PHVD)*.

**Table 2:** **Most promising biomarkers and usefulness in neonatal brain injury**.

Biomarker	Fluids locations	Change	Associations (reference)	Usefulness
S100β	Urine, Blood, CSF	↑	IVH (Gazzolo et al., 1999, 2007, 2010)	++
			Asphyxia and HIE (Gazzolo et al., 2001, 2005)	
Activin A	Blood	↑	IVH (Florio et al., 2006, 2007)	++
			Asphyxia (Florio et al., 2004)	
Epo	Blood	↑	IVH (Bhandari et al., 2011)	++
CCL18	Blood	↓	Lower concentrations in neonates that developed IVH (Kallankari et al., 2010)	++
TGF-β1	CSF	↑	PHVD (Whitelaw et al., 1999, 2001; Lipina et al., 2010)	+
		↑	PHVD patients that that required shunt (Whitelaw et al., 1999)	+++
TGF-β2	CSF	↑	PHVD (Chow et al., 2005)	+
			PHVD patients that develop white mater injury and worse neurodevelopmental outcomes at 15 months (Chow et al., 2005)	+
MMP-9	CSF	↑	PHVD (Okamoto et al., 2008)	+
		↓	Lower concentration in neonates with PHVD that required shunt (Okamoto et al., 2008)	+++
PAI-1	CSF	↑	PHVD (Hansen et al., 2000)	+
			Highest concentration observed in neonates that required shunt (Hansen et al., 2000)	++
GFAP and NFL	CSF	↑	PHVD (Whitelaw et al., 2001)	+

*Table 1 provides a summary of potential biomarkers. These biomarkers have been detected in blood, urine, and cerebrospinal fluid (CSF). Usefulness of the biomarkers: (+) limited use because CSF samples are required, (++) very useful but can be altered by other factors such as gestational age and intrauterine growth restriction, (+++) useful because it may foster different therapy discussion (such shunt placement in patients with PHVD) although required CSF sample*.

## Conflict of Interest Statement

The authors declare that the research was conducted in the absence of any commercial or financial relationships that could be construed as a potential conflict of interest.

## Abbreviations

aEEG, amplitude integrated electroencephalogram; CPK-BB, arain-type creatine phosphokinase; CSF, cerebrospinal fluid; CCL18, chemokine ligand 18; CRP, C-reactive protein; EPO, erythropoietin; GA, gestational age; IVH, intraventricular hemorrhage; MMP-9, matrix metalloproteinase-9; PVL, periventricular leukomalacia; PAI-1, plasminogen activator inhibitor 1; TGF-β1, transforming growth factor beta 1; TGF-β2, transforming growth factor beta-2; UA, uric acid.
